# Development of Quality Indicators for the Ultrasound Department through a Modified Delphi Method

**DOI:** 10.3390/diagnostics13243678

**Published:** 2023-12-15

**Authors:** Aiping Zeng, Yang Gu, Li Ma, Xixi Tao, Luying Gao, Jianchu Li, Hongyan Wang, Yuxin Jiang

**Affiliations:** 1 Department of Ultrasound, Peking Union Medical College Hospital, Chinese Academy of Medical Sciences and Peking Union Medical College, No.1 Shuai Fu Yuan, Dong Cheng District, Beijing 100730, China; 2 National Ultrasound Medical Quality Control Center, Beijing 100730, China

**Keywords:** quality indicator, ultrasound, quality improvement, modified Delphi method

## Abstract

This study aims to establish precise quality indicators for evaluating and enhancing ultrasound performance, employing a methodology based on a comprehensive review of the literature, expert insights, and practical application experiences. We conducted a thorough review of both the domestic and international literature on ultrasound quality control to identify potential indicators. A dedicated team was formed to oversee the complete indicator development process. Utilizing a three-round modified Delphi method, we sought expert opinions through personalized email correspondence. Subsequently, data from diverse hospital indicators were collected to validate and assess feasibility. A novel set of seven indicators was compiled initially, followed by the convening of a 36-member nationally representative expert panel. After three rounds of meticulous revisions, consensus was reached on 13 indicators across three domains. These finalized indicators underwent application in various hospital settings, demonstrating their initial validity and feasibility. The development of thirteen ultrasound quality indicators represents a significant milestone in evaluating ultrasound performance. These indicators empower hospitals to monitor changes in quality effectively, fostering efficient quality management practices.

## 1. Introduction

Imaging services wield substantial influence on patient care across all hospital departments. Ultrasound, recognized for its cost-effectiveness and absence of ionizing radiation, stands as a pervasive diagnostic tool in medical practice, offering broad disease detection capabilities. The escalating demand for ultrasound examinations within public health services and clinical realms underscores the imperative for rigorous quality assurance within ultrasound departments. Moreover, the proficiency of sonographers exhibits significant variability across diverse hospital settings and medical domains, largely contingent upon individual expertise [1]. The absence of standardized protocols for quantitatively assessing ultrasound quality leads to inconsistent examination performance. Consequently, there exists a pressing necessity to formulate precise quality metrics capable of robustly evaluating current ultrasound quality standards and steering enhancements in this field.

Quality indicators serve as pivotal tools catering to the requirements of healthcare performance assessment [2,3,4]. Incorporating these indicators into continuous improvement strategies has the potential to raise the standards of ultrasound services. This integration aims not only to enhance quality and service provisions but also to mitigate healthcare disparities, foster uniformity in quality, and ultimately align with the overarching goal of delivering high-quality healthcare services [5,6].

A large sample [7] assessed the variability of quality and productivity metrics commonly utilized by academic radiology departments and reported some useful indicators such as critical results reporting, relative value unit productivity, emergency department turnaround time, and inpatient imaging turnaround time. Harvey et al. [8] provided examples of performance indicators for radiology, such as compliance with equipment maintenance, technologist-to-scanner ratio, error rates for labeling of images, and staff use rates. Sarwar et al. [9] listed some metrics in radiology, including patient access time, equipment staffing level, equipment idle time, percentage of registered technologists, percentage of complications, etc. While prior studies have established quality indicators beneficial for radiology departments, challenges persist in directly applying these indicators within ultrasound departments. The practicality and feasibility of implementing these indicators in clinical practice within the ultrasound domain present significant hurdles, potentially limiting their seamless integration.

Acknowledging the escalating awareness regarding the adverse impact of ultrasound errors on patient outcomes, the imperative for continual enhancement in ultrasound services remains paramount. This study aims to establish a comprehensive set of pertinent and viable ultrasound quality indicators. By synthesizing insights from the literature, expert consensus, and real-world hospital applications, these indicators are expected to evaluate the current quality landscape and foster improvements in ultrasound performance. The ultimate goal is to ensure precise disease diagnosis and treatment efficacy in the future.

## 2. Materials and Methods

### 2.1. The Development of Indicators

**Figure 1:**
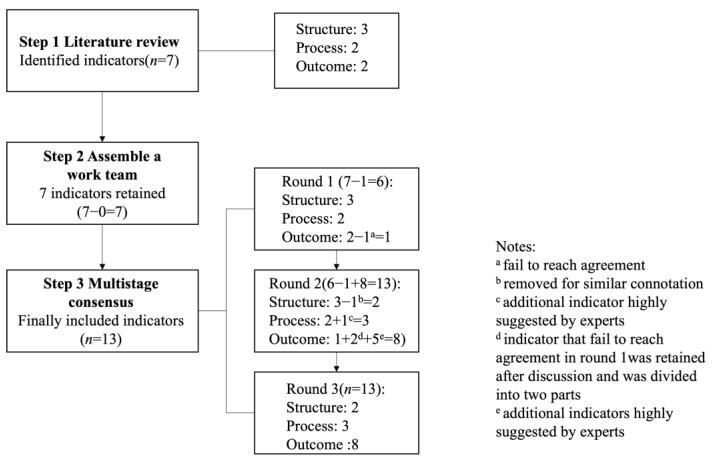
Stages in the development of ultrasound quality indicators.

The process of developing ultrasound quality indicators included a literature review followed by a three-round modified Delphi consensus process (Figure 1).

#### 2.1.1. Step 1: Literature Review

To initiate the process, a comprehensive literature review was undertaken, collating pertinent information on potential indicators from existing benchmarks, quality enhancement endeavors, and relevant documents. Our search strategy encompassed electronic database queries and exploration of gray literature. Databases such as PubMed/MEDLINE, Web of Science, Google Scholar, and the China National Knowledge Infrastructure (CNKI) were meticulously scrutinized to identify pertinent publications.

The gray literature search included books about hospital management and websites that pertained to radiology organizations and organizations that develop and report on quality indicators from China, the USA, and the UK. This process ensured that the first-round questionnaire was evidence-based. In the end, extracted potential indicators would be synthesized into a list employing the Donabedian structure–process–outcome framework [10].

#### 2.1.2. Step 2: Assembling a Work Team

A specialized work team was convened to oversee the development of ultrasound quality indicators. This team comprised ten individuals from the China National Ultrasound Quality Control Centre (NUQCC), an official institution tasked with nationwide ultrasound quality management. The team engaged in a series of in-person conferences to meticulously review the program’s methodology, scrutinize identified indicators from the literature, and subsequently formulate recommended selection criteria. They collaboratively curated a shortlist of candidate indicators to present to the expert panel within the modified Delphi process. Furthermore, the team made minor refinements to certain indicator explanations. These refined indicators formed the basis for constructing the preliminary questionnaire.

#### 2.1.3. Step 3 Multistage Consensus

Subsequently, expert consultation was undertaken to gather professional insights, culminating in consensus via the modified Delphi method. The modified Delphi panel engaged in three rounds of deliberation, comprising two rounds of opinion solicitation through personalized emails utilizing an online survey questionnaire, interspersed with a panel discussion conference.

The selection criteria for the expert panel were as follows: (1) individuals serving as chief or core members within provincial/municipal ultrasound quality centers across China’s 31 provinces, autonomous regions, and municipalities, excluding members of the work team; (2) expertise encompassing various clinical domains of ultrasound practice, including abdomen, breast, cardiac, gynecological, musculoskeletal, obstetrics, pediatric, renal, superficial parts, and vascular areas; (3) a minimum of 15 years of practical experience in ultrasound clinical practice; and (4) proficiency in medical quality management, specifically in supervising, assessing, and managing sonographers’ performance, as well as receiving and responding to patient or referrer feedback. Eligible experts were required to meet all specified criteria.

In the initial round, the expert panel was prompted to assess and classify each indicator. A comprehensive background document detailing the study’s rationale, methodology, a summary of the supporting literature, recommended criteria, and measurement specifications was provided at the outset of the preliminary questionnaire for reference. Each expert was tasked with categorizing each candidate quality indicator into one of three classifications: ‘disagree,’ ‘agree, with comments,’ or ‘agree.’ Furthermore, the questionnaire included a free-text comment field enabling experts to propose additional indicators or offer suggestions regarding the recommended criteria developed by the work team for each indicator.

In the subsequent round (Round 2), the expert panel convened for an in-person conference, focusing on discussion and commentary on candidate indicators. This included indicators that did not achieve consensus in Round 1, along with newly proposed indicators garnering significant attention. Additionally, refinements in phrasing and the provision of clear definitions for indicators were undertaken. Subsequently, a revised questionnaire was formulated based on the outcomes of the preceding two rounds.

Moving to Round 3, the expert panel received the updated questionnaire for a re-evaluation of indicators, akin to the process in Round 1. Indicators that attained at least a 75% consensus rating of ‘agree, with comments’ or ‘agree’ were deemed as accepted.

### 2.2. Analysis of Indicators

To ensure the efficacy and practicality of indicators, mainland Chinese hospitals offering ultrasound diagnosis were invited, in collaboration with local governments and health commissions, to partake in a survey. The survey’s objective was to gather data concerning ultrasound indicators and other pertinent information, collected and submitted on an annual basis. Within each participating hospital, a chief or associate chief doctor from the ultrasound department was assigned as the lead. They received specialized training to acquaint themselves with the indicators, oversee the recording and analysis of ultrasound quality data, and subsequently input this data into the NUQCC database (https://www.nuqcc.cn, accessed on 1 April 2023). The baseline data for ultrasound quality indicators in 2020 was sourced from the NUQCC database.

Concurrently, a national administrative directive initiated a comprehensive ultrasound quality improvement program. Following one year of program implementation, 2021 indicator data were obtained from the NUQCC database, mirroring the prior approach. Comparative analyses between the two years assessed changes in quality indicators. Quantitative data were presented as means, while qualitative data were depicted as frequencies. Normal distribution was tested using the Shapiro–Wilk test. Differences in nonparametric data were evaluated via the Mann–Whitney U test, whereas differences in parametric data were assessed using the paired t-test, considering a significance threshold of *p*-value < 0.05 (SPSS Statistics version 24.0, IBM Corp., Armonk, NY, USA).

## 3. Results

### 3.1. Literature Review

A literature search was executed to pinpoint potentially relevant indicators from medical imaging indicator research and governmental documents focusing on existing medical quality indicators [7,8,9,10,11,12,13,14,15,16,17,18,19,20]. Following a comprehensive review of full-text manuscripts, a total of seven indicators were identified and summarized. These comprised three structure indicators, two process indicators, and two outcome indicators.

### 3.2. Assembling a Work Team

The work team consisted of six ultrasound experts, among whom three held administrative positions, complemented by an experienced medical quality manager, a researcher, a member of the medical service department, and a policy-maker. Collaboratively, the team meticulously reviewed the initial draft indicators, ultimately including a total of seven candidate indicators following an iterative review process (as presented in Table 1). The refinement process involved summarizing references and implementing minor adjustments in wording and explanations.

The work team also devised specific criteria for the expert panels to consider when evaluating and providing feedback on the candidate indicators [21,22,23]: (1) importance: ensuring the indicators encapsulate crucial facets of ultrasound practice, (2) validity: assessing the indicators’ representation within the comprehensive framework, and (3) feasibility: ensuring that the information required for submission is readily accessible and easy to collect.

### 3.3. Multistage Consensus

Thirty-six experts constituted the panel. The response rates in round 1, round 2 and round 3 were 75% (n = 27), 100% (n = 36) and 92% (n = 33), respectively. In round 1, a questionnaire containing seven indicators was presented to the expert panel. Six (86%) indicators (except the positive rate) reached consensus (Table 2). Additionally, we also received opinions and concerns about indicators and other highly proposed indicators.

During Round 2, the expert panel engaged in face-to-face discussion conferences to evaluate the Round 1 results, exchange perspectives, and delve into detailed discussions on any concerns. This forum facilitated further refinement and development of several indicators through comprehensive discussions. Subsequently, following Round 2, a refined set of 13 indicators spanning three domains was finalized. These revised indicators formed the basis of the questionnaire that would be circulated to experts for Round 3 evaluation.

During Round 3, the expert panel reviewed the outcomes of the sample survey embedded within the questionnaire, reassessing the 13 indicators through personalized email correspondence, and applying the same criteria as utilized in Round 1. Table 3 presents the conclusive ratings for each indicator. Following two Delphi rounds and discussion conferences, consensus was achieved on 13 ultrasound performance quality indicators. These encompassed two structure indicators, three process indicators, three general outcome indicators, and five disease-specific outcome indicators, as outlined in Table 4, and further details are shown in the Supplementary Materials.

### 3.4. Applications of Indicators in Chinese Hospitals

The implemented national ultrasound quality improvement program encompassed initiatives such as promoting standardized ultrasound scan protocols, establishing definitions for ultrasound quality indicators, and implementing quality control management standards. This program was executed through various channels, including annual national conferences on ultrasound quality control, web-based training sessions, and quarterly departmental quality control meetings. Notably, the voluntary enrollment count for hospitals participating in the program amounted to 7043 in 2020 and increased to 7095 in 2021.

The average monthly workload per sonographer was 570.30 in 2020 and 623.37 in 2021 (*p* < 0.05). The ultrasound instrument quality inspection rate was 94.65% in 2020 and 97.19% in 2021 (*p* < 0.001). The completion rate of inpatient ultrasound examinations within 48 h was 93.27% in 2020 and 96.33% in 2021 (*p* = 0.015). The qualification rate of ultrasound reports was 96.38% in 2020 and 98.51% in 2021 (*p* = 0.002). The accuracy rate of ultrasound diagnosis of breast lesions was 73.53% in 2020 and 82.46% in 2021 (*p* < 0.001). The incidence of major complications associated with ultrasound-guided interventions was 0.37% in 2020 and 0.89% in 2021 (*p* = 0.001).

Other quality indicators did not show significant differences, as shown in Table 5. The completion rate of notification of ultrasound critical findings within 10 min was 94.89% in 2020 and 97.91% in 2021 (*p* = 0.050). The positive rate of outpatient and emergency ultrasound examinations (2020 vs. 2021: 71.50% vs. 70.59%, *p* = 0.499) was very close. The positive rate of inpatient ultrasound examinations (2020 vs. 2021: 77.76% vs. 76.46%, *p* = 0.421) was very close too. The coincidence rate of ultrasound diagnoses was 73.53% in 2020 and 82.46% in 2021 (*p* = 0.676). The BI-RADS utilization rate for breast lesions in ultrasound reports was 81.78% in 2020 and 79.52% in 2021 (*p* = 0.436). The detection rate of fatal fetal malformations in ultrasound screening for pregnant women was 0.06% in 2020 and 0.07% in 2021 (*p* = 0.149). The analysis of the “coincidence rate of ultrasound diagnosis of ≥50% carotid stenosis” is excluded due to the lack of empirical data.

## 4. Discussion

This study developed a set of ultrasound quality indicators using a modified Delphi method by the national expert panel. Utilizing the anonymous Delphi methodology, which fosters candid responses and consolidates collective expert opinions, has gained widespread acceptance across diverse healthcare domains [24,25,26]. The engagement and feedback from the expert panel underscored the importance of quality assessment and emphasized the necessity for constructing quality indicators to evaluate ultrasound performance.

Ondategui-Parra et al. [11] investigated prevalent management performance indicators in academic radiology departments within the USA, identifying six categories encompassing 28 performance indicators, such as ‘productivity, reporting, access, satisfaction, and finance’. Similarly, Karami et al. [16] developed a comprehensive set of 92 indicators for academic radiology departments using the Delphi method. These indicators were categorized into seven main domains, including ‘safety, service, internal and external customers, teaching and research, resource utilization, financial performance, and workplace excellence’. Our study proposes thirteen quality indicators for ultrasound departments, comprising two structure indicators, three process indicators, three general outcome indicators, and five disease-specific outcome indicators. These indicators hold applicability across various levels, spanning from individual, sectional, and departmental levels to the broader hospital, provincial, and national levels. This broad scope serves to enhance awareness and facilitate quality improvement initiatives. Furthermore, these indicators may facilitate inter-institutional comparisons, track alterations over time, and ascertain the efficacy of implemented actions in driving improvement.

Among two structure indicators, the quality indicator ‘average monthly workload per sonographer’ assesses the adequacy of human resource allocation and organizational structure essential for delivering quality care. Maintaining appropriate staffing levels and manageable workloads is crucial for upholding ultrasound quality [27]. Excessive workloads pose the risk of incomplete studies, emphasizing the importance of not just ‘quantity’ but also ‘quality’ in performance. The quality indicator ‘ultrasound instruments quality inspection rate’ serves as a vital criterion to evaluate ultrasound equipment quality and safety. Malfunctioning or obsolete equipment may compromise image quality. Regular inspection and maintenance are pivotal to ensuring optimal equipment performance, thus safeguarding the production of high-quality images crucial for accurate interpretation [28].

Among the three process indicators, the quality indicator ‘completion rate of inpatient ultrasound examinations within 48 h’ offers valuable insights into the influx of examination requests and the ultrasound department’s capacity to manage them, reflecting the overall accessibility of medical resources. Timely examinations play a crucial role in early diagnosis, preventing patient deterioration, reducing hospital stays, enhancing overall efficiency, and curbing costs [13]. Unlike outpatient settings, where examination timing might align with patient convenience, inpatient examinations prioritize urgency based on clinical necessity. The quality indicator ‘completion rate of notification of ultrasound critical findings within 10 min’ safeguards the prompt reporting of critical ultrasound findings. Critical findings encompass new or unexpected discoveries that could lead to severe morbidity or mortality without appropriate diagnostic or therapeutic interventions [29]. Efficient and timely communication is paramount for patient safety in such instances, while inadequate communication can lead to serious adverse events.

The quality indicator ‘qualification rate of ultrasound reports’ mirrors the quality standards upheld within ultrasound examination reports. A qualified report is characterized by clarity, accuracy, confidence, conciseness, completeness, and consistency [30]. Errors within ultrasound reports, such as missing outpatient or hospitalization numbers, clinical diagnoses, measurement data, sex-related errors, or incorrect orientations, can provoke patient complaints and precipitate medical disputes.

In general outcome indicators, the quality indicator “positive rate of outpatient and emergency ultrasound examinations” and quality indicator “positive rate of inpatient ultrasound examinations” are parameters that should be taken into consideration while assessing the accuracy of ultrasound diagnosis. These rates reflect both the appropriateness of clinicians’ prescription of ultrasound examinations and the accuracy of the results obtained. The positive rate is influenced by not only the pretest probabilities of patients having a disease but also by the proficiency of sonographers and the quality of referrals. Clinicians play a crucial role in understanding examination indications, reducing unnecessary or repetitive tests, and preventing the misuse of medical resources. Simultaneously, sonographers must continually enhance their professional expertise and technical skills to avert false-negative diagnoses arising from incomplete or insufficient scans. It is important to note that hospitals dealing primarily with complex cases might display lower coincidence rates in ultrasound examinations compared to those handling simpler cases. However, it is unjustifiable to infer inferior service quality based solely on this comparison. Consequently, these indicators constitute an indispensable and significant component of ultrasound quality evaluation. Patient preconditions can significantly differ across various sectors and hospitals of distinct tiers, such as ultrasound outcomes in medical check-up centers versus those in emergency departments, where, generally, the positive rate of emergency department ultrasound examinations tends to be higher. The quality indicator ‘coincidence rate of ultrasound diagnoses’ holds significant value in assessing the quality of ultrasound diagnosis, primarily reflecting the diagnostic accuracy within a specified period in an ultrasound department. Incorrect diagnoses, such as misdiagnosing breast cancer as a benign lesion, can lead to delayed treatment, exacerbating the patient’s condition and potentially escalating fatality risks. Hence, it is imperative for sonographers to furnish patients and clinicians with reports that exhibit high accuracy in interpretation.

The disease-specific outcome indicators, tailored for diseases suitable or preferable for ultrasound examinations like breast lesions, vascular diseases, and prenatal screening, garnered significant interest. Consequently, the quality indicators ‘BI-RADS utilization rate for breast lesions in ultrasound reports,’ ‘accuracy rate of ultrasound diagnosis of breast lesions,’ ‘detection rate of fatal fetal malformations in ultrasound screening for pregnant women,’ ‘coincidence rate of ultrasound diagnosis of ≥50% carotid stenosis,’ and ‘incidence of major complications associated with ultrasound-guided interventions’ attained consensus. The corresponding definitions for these indicators are delineated in Table 4. These disease-specific outcome indicators offer a more detailed reflection of ultrasound quality and pinpoint specific avenues for directing our quality improvement efforts. Nevertheless, we regard our study as an inaugural phase in an ongoing progression. It is evident that additional quality indicators reflecting the performance of ultrasound across various domains need development and practical implementation to enrich the field and enhance the standard of ultrasound practice.

The data analysis of quality indicators in nationwide hospital implementation demonstrated their feasibility and interpretability to a certain extent. The rise in average monthly workload per sonographer highlighted an escalating demand for ultrasound examinations, signaling the necessity for further investigations to establish optimal staff allocation. This pursuit aims to achieve rational human resource utilization and avert performance issues arising from excessive workloads. The increased ultrasound instruments’ quality inspection rate suggested the timely replacement of equipment that failed quality control tests, and indicated a proactive approach to replacing equipment failing quality control assessments. This contributes significantly to governing ultrasound equipment usage, monitoring image quality, and curbing errors linked to inadequate images. The significant enhancement in the accuracy rate of ultrasound diagnosis of breast lesions indicated improved adherence to standardized ultrasound examination protocols by sonographers. It also signified an enhanced capability to make precise diagnoses after the quality improvement program. The increased qualification rate of ultrasound reports suggested a decline in errors within reports, emphasizing the importance of objectively detailing ultrasound examination information to facilitate effective communication. The improvement in the completion rate of inpatient ultrasound examinations within 48 h suggested an augmented capacity among sonographers, even amidst increased workload demands, resulting in reduced patient waiting times.

Additionally, the changes in the incidence of major complications associated with ultrasound-guided interventions pointed out that corresponding quality improvement programs for ultrasound-guided interventions should be considered to ensure patient safety in the future. The little significance of other quality indicators (including completion rate of notification of ultrasound critical findings within 10 min, detection rate of fatal fetal malformations in ultrasound screening for pregnant women, BI-RADS utilization rate for breast lesions in ultrasound reports, positive rate of outpatient and emergency ultrasound examinations, positive rate of inpatient ultrasound examinations, and coincidence rate of ultrasound diagnoses) can be explained by subtle changes in the pretest probability of corresponding disease prevalence or limited progression in relative skills of sonographers produced by the former quality improvement program. This also reminded us that underlying reasons and better improvement methods need to be further studied in the future.

It is intriguing to compare the findings of Tao et al. [31], who utilized eight quality indicators to assess the impact of a national ultrasound quality improvement program, with our study’s outcomes. Our study aimed to develop ultrasound control indicators, whereas their research focused on implementing a quality improvement program and evaluating its effectiveness. Meanwhile, their results indicated that the program led to improved accuracy in ultrasound diagnosis and highlighted an increasing demand for ultrasound examinations, signaling the necessity for more ultrasound practitioners. In contrast, our study, focusing on disease-specific quality indicators, specifically identifies the areas where ultrasound diagnosis accuracy has been enhanced. Furthermore, the changes observed in process indicators offer insights into specific workflow areas that necessitate improvement. This distinction in focus between disease-specific indicators and process indicators provides a more nuanced understanding of the improvements resulting from the quality improvement program. Both approaches, Tao et al.’s broader assessment and our study’s specific focus, contribute to comprehensively evaluating the impact and areas for refinement in ultrasound quality initiatives.

Thus, the results provide some evidence, and it can be preliminarily judged that these quality indicators are likely to monitor changes in ultrasound quality, reflect meaningful information about deficiencies, and guide quality improvement measures to improve quality when poor performance is observed. Nevertheless, this study analyzed the application of the quality indicators over a 2-year period, which may not be long enough to completely validate whether changes in indicators reflect true changes in quality. For participating hospitals, how the data are interpreted and how data interpretation can lead to quality improvement are of major concern. Further approaches to address the validity of the data, evaluate the feasibility of data collection and sensitivity to change, and examine discriminatory power are warranted in future implementations.

While this study demonstrates the first effort to develop quality indicators for the ultrasound department, there are some limitations. First, there is a lack of indicators of research and education, costs, and the value of other stakeholders such as patients and referring physicians. Future efforts should focus on drilling down to obtain further detail. Second, our study demonstrates the establishment of ultrasound quality control indicators and the initial results of the application of the indicators. The optimal and suboptimal threshold of agreed-upon indicators need to be determined in further research. Third, this is a study based on national but not international experts. We also look forward to future collaborations with international experts to establish quality indicators that are broadly applicable to ultrasound medicine in most countries.

## 5. Conclusions

Our study developed 13 ultrasound quality indicators through a literature review and national expert consensus-building. Implementation across multiple hospitals nationwide demonstrated that 13 ultrasound quality indicators can effectively accomplish continuous data collection and faster assessment. These ultrasound quality indicators serve as a valuable framework for assessing ultrasound practice, empowering hospitals and sonographers to deliver high-quality healthcare services. It also encourages more effective management strategies, paving the way for improved healthcare standards in the future.

## Figures and Tables

**Table 1:** Candidate ultrasound quality indicators in the round 1 questionnaire.

Candidate Ultrasound Quality Indicators
Structure indicators Daily workload in outpatient, emergency, physical examination, and inpatient departments.
2. Ratio of sonographers to patients in the ultrasound department. 3. Ratio of sonographers to equipment. Process indicators 4. Average appointment waiting time in the inpatient department. 5. Number of critical results reporting. Outcome indicators 6. Positive rate of all reports. 7. Coincidence rate of ultrasound diagnosis.

**Table 2:** Rating results of the Delphi round 1.

Indicator	Disagree	Agree, with Comments	Agree	Consensus ^1^	% ^2^
1	3	1	23	24/27	0.89
2	5	3	19	22/27	0.81
3	4	4	19	23/27	0.85
4	3	3	21	24/27	0.89
5	4	10	13	23/27	0.85
6	7	6	14	20/27	0.74
7	2	2	23	25/27	0.93

^1^ Consensus: number of ‘agree, with comments’ and ‘agree’/total number of responses; ^2^ %: percentage of ‘agree, with comments’ and ‘agree’ among all responses.

**Table 3:** Rating results of the Delphi round 3.

Indicator	Disagree	Agree, with Comments	Agree	Consensus ^1^	% ^2^
1	5	15	13	28/33	0.85
2	4	6	23	29/33	0.88
3	2	9	22	31/33	0.94
4	5	14	14	28/33	0.85
5	1	4	28	32/33	0.97
6	3	8	22	30/33	0.91
7	2	8	23	31/33	0.94
8	1	6	26	32/33	0.97
9	5	8	20	28/33	0.85
10	5	12	16	28/33	0.85
11	1	2	30	32/33	0.97
12	2	7	24	31/33	0.94
13	2	6	25	31/33	0.94

^1^ Consensus: number of ‘agree, with comments’ and ‘agree’/total number of responses; ^2^ %: percentage of ‘agree, with comments’ and ‘agree’ among all responses.

**Table 4:** Final set of ultrasound quality indicators.

Indicator	Definition
Structure indicators	
1. Average monthly workload per sonographer	the average number of ultrasound reports issued by each sonographer per month
2. Ultrasound instruments quality inspection rate (%)	the proportion of the number of ultrasound instruments passed the quality inspections among the total number of ultrasound instruments in the ultrasound department during the same period
Process indicators	
3. Completion rate of inpatient ultrasound examinations within 48 h (%)	the proportion of the number of inpatient ultrasound examinations completed within 48 h of clinical requests among the total number of inpatient ultrasound examination requests issued by the clinic during the same period
4. Completion rate of notification of ultrasound critical findings within 10 min (%)	the proportion of the number of ultrasound examinations with critical findings reported to clinical doctors within 10 min among the total number of ultrasound examinations with critical findings during the same period
5. Qualification rate of ultrasound reports (%)	the proportion of the number of qualified ultrasound reports among the total number of ultrasound reports during the same period
General outcome indicators	
6. Positive rate of outpatient and emergency ultrasound examinations (%)	the proportion of the number of outpatient and emergency ultrasound examinations with any positive findings among the total number of ultrasound examinations during the same period
7. Positive rate of inpatient ultrasound examinations (%)	the proportion of the number of inpatient ultrasound examinations with any positive findings among the total number of ultrasound examinations during the same period
8. Coincidence rate of ultrasound diagnoses (%)	the proportion of the number of ultrasound diagnoses consistent with pathological or clinical diagnoses among the total number of ultrasound diagnoses with corresponding pathological or clinical diagnoses during the same period
Disease-specific outcome indicators	
9. Breast Imaging Reporting and Database System (BI-RADS) utilization rate for breast lesions in ultrasound reports (%)	the proportion of the number of ultrasound reports of breast lesions using the BI-RADS template among the total number of ultrasound reports of breast lesions during the same period
10. Accuracy rate of ultrasound diagnosis of breast lesions (%)	the proportion of the number of breast ultrasound diagnosed as breast cancers or non-breast cancers consistent with pathological results among the total number of ultrasound diagnoses of breast lesions with corresponding pathological results during the same period
11. Detection rate of fatal fetal malformations in ultrasound screening for pregnant women (%)	the proportion of the number of pregnant women with fatal fetal malformations detected in ultrasound obstetric screening among the total number of pregnant women with ultrasound obstetric screening during the same period
12. Coincidence rate of ultrasound diagnosis of ≥50% carotid stenosis (%)	the proportion of the number of ultrasound diagnoses of carotid stenosis (≥50%) that is consistent with other imaging results such as DSA or CTA among the total number of ultrasound diagnoses of carotid stenosis (≥50%) with other imaging results available such as DSA or CTA during the same period
13. Incidence of major complications associated with ultrasound-guided interventions (%)	the proportion of the number of major complications associated with ultrasound-guided interventions among the total number of ultrasound-guided interventions during the same period

**Table 5:** Performance assessment of ultrasound quality indicator in 2020 and 2021.

Quality Indicator	2020	2021	*p* Value
1. Average monthly work-load per sonographer	570.30	623.37	<0.001
2. Ultrasound instruments quality inspection rate (%)	94.65	97.19	0.001
3. Completion rate of inpatient ultrasound examinations within 48 h (%)	93.27	96.33	0.015
4. Completion rate of notification of ultrasound critical findings within 10 min (%)	94.89	97.91	0.050
5. Qualification rate of ultrasound reports (%)	96.38	98.51	0.002
6. Positive rate of outpatient and emergency ultrasound examinations (%)	71.50	70.59	0.499
7. Positive rate of inpatient ultrasound examinations (%)	77.76	76.46	0.421
8. Coincidence rate of ultrasound diagnoses (%)	84.75	85.40	0.676
9. Breast Imaging Reporting and Database System (BI-RADS) utilization rate for breast lesions in ultrasound reports (%)	81.78	79.52	0.436
10. Accuracy rate of ultrasound diagnosis of breast lesions (%)	73.53	82.46	<0.001
11. Detection rate of fatal fetal malformations in ultrasound screening for pregnant women (%)	0.06	0.07	0.149
12. Coincidence rate of ultrasound diagnosis of ≥50% carotid stenosis (%)	0.37	0.89	0.001

## Data Availability

The authors declare that they had full access to all of the data in this study, and take complete responsibility for the integrity of the data and the accuracy of the data analysis.

## Supplementary Materials

The following supporting information can be downloaded at: https://www.mdpi.com/article/10.3390/diagnostics13243678/s1.
