# Supplementary material for: Development of Quality Indicators for the Ultrasound Department through a Modified Delphi Method
Source: Diagnostics (Basel). 2023 Dec 15;13(24):3678. doi: 10.3390/diagnostics13243678 (PMC10743281; doi:10.3390/diagnostics13243678)
Supplement: Supplementary file 1 [file diagnostics-13-03678-s001.zip › diagnostics-2711005-supplementary.pdf]

Supplementary materials

Definitions, formula and explanations of thirteen ultrasound quality indicators

Indicator 1

Average monthly workload per sonographer

Definition: the average number of ultrasound reports issued by each sonographer per month.

Formula:

$$\frac{\text{the total annual workload of ultrasound department}}{\text{the number of sonographers} \times \text{12 months}}$$

Explanation: 1) The total annual workload of ultrasound department refers to the total number of ultrasound reports issued by sonographers. 2) The sonographer refers to the doctor who has obtained the 'Physician's Practice Certificate' and is engaged in ultrasound diagnosis and treatment full-time in the institution and works for at least 6 months per year.

Indicator 2

Ultrasound instruments quality inspection rate (%)

Definition: the proportion of the number of ultrasound instruments passed the quality inspections among the total number of ultrasound instruments in the ultrasound department during the same period.

Formula:

$$\frac{\text{the number of ultrasound instruments passed the quality inspections}}{\text{the total number of ultrasound instruments in the ultrasound department during the same period}}$$

Explanation: Ultrasound instruments quality inspection refers to the annual measurement and imaging quality inspection conducted by the nationally recognized measurement and testing institutions.

Indicator 3

Completion rate of inpatient ultrasound examinations within 48 hours (%)

Definition: the proportion of the number of inpatient ultrasound examinations completed within 48 hours of clinical requests among the total number of inpatient ultrasound examinations requests issued by the clinic during the same period.

Formula:

$$\frac{\text{the number of ultrasound examinations completed within 48 hours of clinical requests}}{\text{the total number of inpatient ultrasound examinations requests issued by the clinic during the same period}}$$

Indicator 4

Completion rate of notification of ultrasound critical findings within 10 minutes (%)

Definition: the proportion of the number of ultrasound examinations with critical findings reported to clinical doctors within 10 minutes among the total number of ultrasound examinations with critical findings during the same period.

Formula:

$$\frac{\text{the number of ultrasound examinations with critical findings reported to clinical doctors within 10 minutes}}{\text{the total number of ultrasound critical findings during the same period}}$$

Explanation: Critical findings including: 1) suspected rupture and hemorrhage of liver, spleen and kidney, 2) suspected rupture of ectopic pregnancy with intraperitoneal hemorrhage, 3) the suppurated with acute perforation gallbladder in acute cholecystitis, 4) oligohydramnios and fetal heart rate are too fast (>160 beats/min) or too slow (<110 beats/min) in late pregnancy, 5) uterine rupture, 6) placental abruption, 7) placenta previa and active bleeding, 8) first detection of cardiac dysfunction (LVEF<35%), 9) pericardial effusion with cardiac compression, 10) aortic

dissection, 11) rupture of aortic aneurysm, 12) cardiac rupture, 13) free thrombus of heart, 14) acute arterial embolism of upper and lower limbs, and 15) valve sticking after valve replacement.

Indicator 5

Qualification rate of ultrasound reports (%)

Definition: the proportion of the number of qualified ultrasound reports among the total number of ultrasound reports during the same period.

Formula:

$$\frac{\text{the number of qualified ultrasound reports}}{\text{the total number of ultrasound reports during the same period}}$$

Explanation:

With one of the following conditions is considered an unqualified ultrasound report:

1) ultrasound reports without the signature of a qualified sonographer;

2) not containing the examination of the items prescribed in the application form;

3) where the description in the ultrasound report is inconsistent with the conclusion;

4) ultrasound reports with obvious errors, including: the organs examined are missing but reported as normal; errors in the orientation (left and right, up and down, front and back), units of measurement and data of the examined organs, parts, and lesions described in the ultrasound report; failure to remove template text that is ambiguous with the ultrasound report; the name, gender, and hospitalization number of the patient in the ultrasound report do not match the actual situation.

Indicator 6

Positive rate of outpatient and emergency ultrasound examinations (%)

Definition: the proportion of the number of outpatient and emergency ultrasound examinations with any positive findings among the total number of ultrasound examinations during the same period.

Formula:

$$\frac{\text{the number of ultrasound examinations with positive findings}}{\text{the total number of outpatient and emergency ultrasound examinations during the same period}}$$

Explanation: 1) This indicator is calculated by the number of reports. If a report contains multiple examination sites with one positive or multiple positive results, it is counted as one positive report.

2) This indicator does not include physical examination ultrasound reports.

Indicator 7

Positive rate of inpatient ultrasound examinations (%)

Definition: the proportion of the number of inpatient ultrasound examinations with any positive findings among the total number of ultrasound examinations during the same period.

Formula:

$$\frac{\text{the number of ultrasound examinations with positive findings}}{\text{the total number of inpatient ultrasound examinations during the same period}}$$

Explanation: The same as indicator 6.

Indicator 8

Coincidence rate of ultrasound diagnoses (%)

Definition: the proportion of the number of ultrasound diagnoses consistent with pathological or clinical diagnoses among the total number of ultrasound diagnoses with corresponding pathological or clinical diagnoses during the same period.

Formula:

$$\frac{\text{the number of ultrasound diagnoses consistent with pathological or clinical diagnoses}}{\text{the total number of ultrasound diagnoses with corresponding pathological or clinical diagnosis}}$$

Explanation:

1) Only the number of cases in which the ultrasound diagnosis had a corresponding pathological diagnosis or final clinical diagnosis was counted.

2) The diagnostic criteria were determined by surgical diagnosis or postoperative pathological diagnosis, clinical test indexes, dynamic follow-up outcomes, other supporting imaging examinations and case discussions, and comprehensive analysis.

Indicator 9

Breast Imaging Reporting and Database System (BI-RADS) utilization rate of breast lesions in ultrasound reports (%)

Definition: the proportion of the number of the ultrasound reports of breast lesions using BI – RADS template among the total number of ultrasound reports of breast lesions during the same period.

Formula:

$$\frac{\text{the number of the ultrasound reports of breast lesions using BI} - \text{RADS template}}{\text{the total number of ultrasound reports of breast lesions during the same period}}$$

Indicator 10

Accuracy rate of ultrasound diagnosis of breast lesions (%)

Definition: the proportion of the number of breast ultrasound diagnosed as breast cancers or non-breast cancers consistent with pathological results among the total number of ultrasound diagnosis of breast lesions with corresponding pathological results during the same period.

Formula:

$$\frac{\begin{array}{c}\text{the number of breast ultrasound diagnosed as breast cancers or non} - \text{breast cancers} \\ \text{consistent with pathological results}\end{array}}{\begin{array}{c}\text{the total number of ultrasound diagnosis of breast lesions} \\ \text{with corresponding pathological results during the same period}\end{array}}$$

Explanation: 1) True positives and true negatives refer to ACR BI-RADS® 2013

Ultrasound	Biopsy results	
	Positive (breast cancer diagnosed within 1 year by histology)	Negative (benign on biopsy or no malignant tumor found within 1 year)
Positive （BI-RADS Category 4,5）	True Positive	False Positive
Negative （BI-RADS Category 1,2,3）	False Negative	True Negative

2) Cases underwent breast ultrasound examination before biopsy or surgical excision with confirmed pathology; cases with unclear pathological diagnosis were excluded.

3) The final pathologic results were considered the diagnostic gold standard.

Indicator 11

Detection rate of fatal fetal malformations in ultrasound screening for pregnant women (%)

Definition: the proportion of the number of pregnant women with fatal fetal malformations detected in ultrasound obstetric screening among the total number of pregnant women with ultrasound obstetric screening during the same period.

Formula:

$$\frac{\text{the number of pregnant women with fatal fetal malformations detected in ultrasound obstetric screening}}{\text{total number of pregnant women with ultrasound obstetric screening during the same period}}$$

Explanation: 1) The major fatal fetal malformations include anencephaly, severe encephalocele, severe open spina bifida, severe thoracoabdominal wall defects, visceral ectropion, single lumen heart, and fatal chondrodysplasia.

2) The statistics of this indicator are calculated according to the number of pregnant women. Multiple ultrasound examinations of the same pregnant woman (including multiple fetuses) will be counted as one visit.

3) This indicator is only applicable to medical institutions that provide maternity services.

Indicator 12

Coincidence rate of ultrasound diagnosis of ≥ 50% carotid stenosis (%)

Definition: the proportion of the number of ultrasound diagnosis of carotid stenosis (≥ 50%) that consistent with other imaging results such as DSA or CTA among the total number of ultrasound diagnosis of carotid stenosis (≥ 50%) with other imaging results available such as DSA or CTA during the same period.

Formula:

$$\frac{\begin{array}{c}\text{the number of ultrasound diagnosis of carotid stenosis } (\geq\ 50\%)\\ \text{that consistent with other imaging results such as DSA or CTA}\end{array}}{\begin{array}{c}\text{the total number of ultrasound diagnosis of carotid stenosis } (\geq\ 50\%)\\ \text{with other imaging results available such as DSA or CTA during the same period}\end{array}}$$

Explanation: the lateralization, name, and degree of ultrasound carotid stenosis should be consistent with other imaging results such as DSA or CTA.

Indicator 13

Incidence of major complications associated with ultrasound-guided interventions (%)

Definition: the proportion of the number of major complications associated with ultrasound-guided interventions among the total number of ultrasound-guided interventions during the same period.

Formula:

$$\frac{\text{the number of major complications associated with ultrasound} - \text{guided interventions}}{\text{the total number of ultrasound} - \text{guided interventions during the same period}}$$

Explanation: 1) Ultrasound-guided interventions include ultrasound-guided diagnostic and therapeutic interventions such as biopsy, aspiration, drainage, intubation, drug injection therapy, and ablation.

2) The major complications include bleeding, infection, injury of adjacent organs, nerve injury, needle implantation, etc.
